# Exploring the role of mitophagy-related genes in breast cancer: subtype classification and prognosis prediction

**DOI:** 10.7150/ijms.100785

**Published:** 2024-10-14

**Authors:** Lizhao Wang, Nan Mei, Jianpeng Li, Heyan Chen, Jianjun He, Ru Wang

**Affiliations:** 1 Department of Breast Surgery, The First Affiliated Hospital of Xi'an Jiaotong University, Xi'an, Shaanxi, 710061, China.; 2 Department of Hematology, The First Affiliated Hospital of Xi'an Jiaotong University, Xi'an, Shaanxi, 710061, China.; 3 Department of Urology, The First Affiliated Hospital of Xi'an Jiaotong University, Xi'an, Shaanxi, 710061, China.

**Keywords:** breast cancer, mitophagy, prognosis, immune, gene signature

## Abstract

**Background:** Breast cancer (BC) is the most common cancer among women globally and poses the leading health threat to women worldwide, with persistently high incidence rates. Mitophagy is a selective autophagy process that specifically targets mitochondria within the cell, maintaining cellular energy balance and metabolic health by identifying and degrading damaged mitochondria. Although there is an understanding of the relationship between mitophagy and cancer, the specific mechanisms remain unclear due to the complexity and diversity of mitophagy, suggesting that it could be an effective and more targeted therapeutic approach for BC.

**Methods:** In this study, we meticulously examined the BC expression and clinical pathology data from The Cancer Genome Atlas (TCGA) to assess the expression profiles, copy number variations (CNV), and to investigate the correlation, function, and prognostic impact of 34 mitophagy-related genes (MRGs). Differentially expressed genes (DEGs) were identified based on group classification. Lasso and Cox regression were used to determine prognostic genes for constructing a nomogram. Single-cell analysis mapped the distribution of these genes in BC cells. Additionally, the association between gene-derived risk scores and factors such as immune infiltration, tumor mutational burden (TMB), cancer stem cell (CSC) index, and drug responses was studied. *In vitro* experiments were conducted to confirm the analyses.

**Results:** We included 34 MRGs and subsequently generated a risk score for 7 genes, including RPLP2, PCDHGA2, PRKAA2, CLIC6, FLT3, CHI3L1, and IYD. It was found that the low-risk group had better overall survival (OS) in BC, higher immune scores, but lower tumor mutational burden (TMB) and cancer stem cell (CSC) index, as well as lower IC50 values for commonly used drugs. To enhance clinical applicability, age and staging were incorporated into the risk score, and a more comprehensive nomogram was constructed to predict OS. This nomogram was validated and showed good predictive performance, with area under the curve (AUC) values for 1-year, 3-year, and 5-year OS of 0.895, 0.765, and 0.728, respectively.

**Conclusion:** Our findings underscore the profound impact of prognostic genes on the immune response and prognostic outcomes in BC, indicating that they can provide new avenues for personalized BC treatment and potentially improve clinical outcomes.

## Introduction

Breast cancer, the leading health threat to women worldwide, maintains a high incidence rate with the unfortunate statistic that one in eight women is affected, making it the primary cause of cancer-related deaths among females^1^,^2^. The essence of the disease lies in the uncontrolled proliferation and growth of aberrant cells within the mammary tissue, which exhibit a complex and mutable nature. At the molecular level, breast cancer, particularly BRCA, demonstrates a high degree of heterogeneity, closely linked to therapeutic resistance^3^. Currently, pathological classification combined with the status of molecular markers such as estrogen receptor (ER), progesterone receptor (PR), and human epidermal growth factor receptor 2 (HER2), delineates breast cancer into subtypes: luminal A, luminal B, basal-like, and HER2-enriched^4^. Despite the current medical system's comprehensive treatment strategies, which include mastectomy, radiation therapy, chemotherapy, endocrine therapy, and targeted therapy, the prognosis for patients with advanced stages remains unfavorable^5^. Hence, there is a necessity to explore new subtypes or biomarkers based on advancements in biological markers and mechanisms, to refine personalized treatment strategies and offer patients improved therapeutic outcomes and a greater hope for survival.

Autophagy is a crucial cellular process that facilitates the degradation and recycling of damaged or unnecessary organelles and proteins within the cell^6^, ensuring its health and functionality, particularly during nutrient deprivation or under stress^7^. Mitophagy, a specialized form of autophagy, plays a pivotal role in maintaining energy balance and metabolic health by selectively targeting and degrading damaged mitochondria^8^,^9^. This process begins with the recognition of dysfunctional mitochondria, which are then sequestered within autophagosomes. These vesicles merge with lysosomes to form autolysosomes, where lysosomal enzymes break down the mitochondrial components. Mitophagy not only removes impaired mitochondria but also recycles certain elements, which is essential for cellular well-being^10^,^11^. Disruptions in this process are linked to a spectrum of diseases, including cancer and neurodegenerative conditions^12^-^14^. The regulation of mitophagy is governed by various signaling pathways and proteins, such as AMP-activated protein kinase (AMPK), mechanistic target of rapamycin (mTOR), and Unc-51-like autophagy-activating kinase 1 (ULK1)^15^-^17^. A thorough understanding of this regulatory mechanism could pave the way for innovative therapeutic strategies for diseases associated with mitochondrial dysfunction and cancer.

Current studies have shown that mitophagy is related to the occurrence and development of tumors^18^,^19^. In breast cancer cells, mitophagy can be impaired by factors such as oxidative stress and metabolic disruptions, which may result in cellular dysfunction and could potentially promote tumor development^20^. The activation of mitophagy could be beneficial in clearing damaged mitochondria, thus reducing the viability and proliferation of tumor cells^21^. Consequently, agents that activate or enhance mitophagy might present a new therapeutic strategy for breast cancer. For instance, research is exploring the potential of mitophagy inducers in treatment^22^. The relationship between mitophagy and cancer, while recognized, remains intricate and enigmatic due to the complexity and diversity of mitophagy's roles in different cancers, particularly BC.

In this study, we downloaded the expression data and corresponding clinical information from breast cancer patients in The Cancer Genome Atlas (TCGA) database. We analyzed the expression profiles of mitophagy-related genes (MRGs) and categorized samples into two distinct mitophagy subtypes based on MRGs expression levels. Subsequently, we identified differentially expressed genes (DEGs) based on these two mitophagy genetic subtypes and stratified patients into three genetic subtypes. Utilizing these DEGs, we constructed and validated a prognostic risk model comprising seven genes, aiming to accurately predict the prognosis of BC. Additionally, we conducted a comprehensive analysis of their distribution in tissues, the immune microenvironment within breast cancer, and drug sensitivity to provide more comprehensive guidance for the treatment and prognosis of breast cancer.

## Methods

### Breast cancer data source and pre-processing

**Figure 1:**
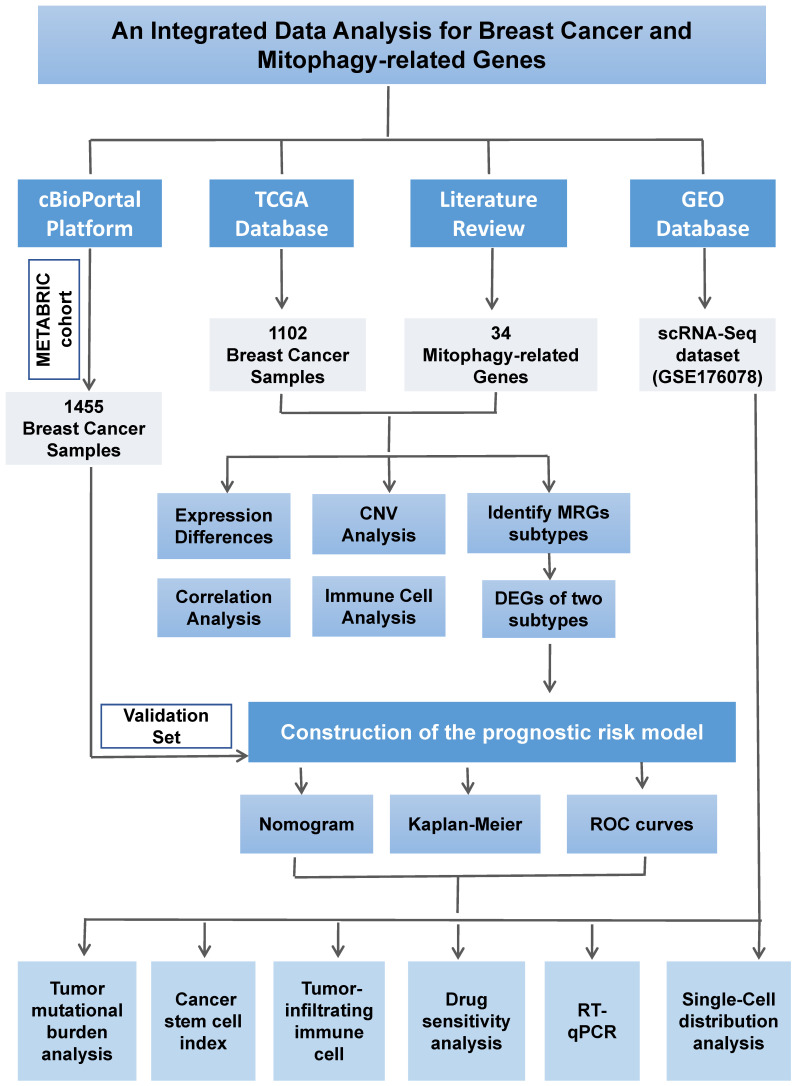
Flow chart of the study design.

The detailed process of this study is illustrated in **Figure 1**. We systematically conducted data collection and analysis. Initially, we utilized the authoritative Cancer Genome Atlas (TCGA) database (https://portal.gdc.cancer.gov/), from which we extracted the mRNA expression profiles and copy number variation (CNV) data of breast cancer (BC) samples, along with detailed clinical and pathological information. To ensure the breadth and representativeness of the data, we meticulously selected 1,102 cases from the TCGA database that had complete follow-up information and clinical pathological data. To further validate our research findings, we also selected 1,455 breast cancer samples with complete prognostic information from the METABRIC cohort within the cBioportal platform (http://www.cbioportal.org) as an independent validation set. This additional data enhances the reliability of our study. Furthermore, to delve into the gene expression characteristics of breast cancer, we downloaded relevant gene expression data from the Gene Expression Omnibus (GEO) database (https://www.ncbi.nlm.nih.gov/geo/).

### Mitophagy-related genes CNVs, and consensus clustering analysis

Based on a review of prior literature, we identified 34 mitophagy-related genes and summarized their details in **Table S1**^19^,^23^-^25^. We visualized the positions of mitophagy-related genes on the 23 chromosomes using the R packages "maftools" and "RCircos." Subsequently, we performed consensus clustering analysis with these genes and employed the "ConsensusClusterPlus" R package to stratify the samples into two distinct mitophagy gene regulatory subtypes. To evaluate the overall survival (OS) of different subtypes, we conducted survival analysis using the R package "survival." Finally, we further analyzed the differentially expressed genes (DEGs) of these subtypes using the "Limma" R package. This integrated approach has facilitated the characterization of the molecular heterogeneity of breast cancer and its impact on patient prognosis and therapeutic strategies.

### GSVA, PCA, and functional enrichment analysis

In this study, we initially employed the Gene Set Variation Analysis (GSVA) approach to meticulously examine and compare the differences in biological processes between distinct mitophagy regulatory gene subtypes. This method is capable of revealing unique patterns of biological pathways and gene expression across subtypes, thereby deepening our understanding of the complexity of breast cancer. Subsequently, to evaluate the immune microenvironment of different mitophagy subtypes in breast cancer, we utilized the single-sample gene set enrichment analysis (ssGSEA) algorithm. This algorithm quantitatively assesses the enrichment of specific gene sets within each sample, aiding in our understanding of immune cell infiltration across different mitophagy subtypes and subsequently analyzing their potential impact on tumor progression and patient prognosis. Furthermore, to achieve a more accurate classification of breast cancer samples, we applied Principal Component Analysis (PCA). PCA is a powerful statistical tool that can reduce high-dimensional gene expression data into several principal components, which capture most of the variation in the data and are used for classification and comparison between samples. To further elucidate the biological functions of MRGs in breast cancer, we conducted Gene Ontology (GO) and Kyoto Encyclopedia of Genes and Genomes (KEGG) pathway analysis. These analyses provide detailed information on the biological processes and metabolic pathways in which genes are involved. Throughout the analysis, we made full use of multiple packages within the R language, including "GSEABase," "clusterProfiler," "GSVA," "limma," "org.Hs.e.g.db," and "enrichplot," among others.

### Construction of the mitophagy-related scoring system and prognostic risk model

We initiated our study with univariate Cox regression to identify prognostic-relevant genes (PRGs) with P < 0.05. Using PRG expression, unsupervised clustering categorized samples into gene clusters, revealing OS differences. The LASSO model refined gene selection, identifying a prognostic gene subset. Multivariate stepwise Cox regression on these genes constructed a mitophagy-related scoring system. The formula for calculating each patient's risk score was:

Risk score = (coefficient 1 × value 1) + (coefficient n × value n)

Where value represents the z-score converted expression of selected genes. We used the "rms" R package to create a nomogram correlating risk scores with clinical features, enhancing our understanding of their combined prognostic impact. The AUC from the ROC curve assessed the model's discriminative ability, with values closer to 1 indicating greater predictive strength. Patients were divided into low- and high-risk groups based on the median risk score. The "dplyr" R package generated a Sankey diagram illustrating the progression from cluster analysis to risk stratification and survival outcomes.

### Single-cell distribution analysis

Our analysis of gene distribution in breast cancer specimens from the scRNA-Seq dataset GSE176078 involved the following steps: Firstly, we utilized the detailed cellular annotations from data contributors for precise cell identification in the initial sequencing, forming the basis for accurately discerning cell types within the dataset. Next, we applied t-SNE and UMAP techniques to visually depict the cellular clusters present. These methods dimensionality reduce high-dimensional gene expression data to 2D or 3D, facilitating intuitive observation of cluster distributions and boundaries. Following cluster identification, we used violin plots to analyze the expression distribution of prognostic genes. These plots, integrating boxplot and kernel density estimation features, illustrated gene expression medians, interquartile ranges, and overall distribution shapes, highlighting expression differences of prognostic genes across clusters and bolstering subsequent biological analyses.

### Tumor immune and cancer stem cell (CSC) index analysis

We assessed tumor-infiltrating immune cells using the CIBERSORT algorithm, which estimates cell type proportions from gene expression data, providing insights into the TME's immune cell landscape, including T cells, B cells, NK cells, DCs, macrophages, and MDSCs. Using the "Estimate" R package, we calculated TME scores to evaluate stromal and immune cell infiltration levels, distinguishing between stromal and immune scores indicative of cellular content and infiltration degree, respectively. Patients were stratified by risk scores derived from mitophagy-related analyses into high- and low-risk groups, and TME score differences were compared to discern TME characteristic variations. Additionally, the correlation between risk scores and stemness scores, which measure tumor cell stemness and correlate with malignancy and relapse, was explored to understand the link between immune cell infiltration and tumor stemness.

### Tumor mutation and drug sensitivity analysis

In this study, we extracted somatic mutation data of breast cancer from the TCGA repository and utilized the "maftools" R package to convert it into the MAF format. We subsequently analyzed the mutation profiles of samples in both high- and low-risk cohorts and quantified their tumor mutational burden (TMB) scores. Further exploration was conducted to investigate the potential correlation between TMB scores and risk scores. Ultimately, using the "pRophetic" R package, we calculated the IC50 values of commonly used chemotherapeutic agents in the management of breast cancer to compare the therapeutic response to chemotherapy across different risk stratifications.

### RT-qPCR

Utilizing the RNA fast 200 RNA Extraction kit from Fastagen Biotech (Shanghai, 220010), we isolated total RNA from both normal mammary epithelial cells (MCF-10A) and breast cancer (BC) cell lines, including HCC-1954, SUM 159, T-47D, and MDA-MB-231. Subsequently, cDNA synthesis was performed employing the StarScript III RT MasterMix provided by Genestar. The quantification of mRNA expression levels was achieved through the application of SYBR-Green assays, procured from Abclonal, and conducted on a Bio-Rad CFX-96 instrument (Hercules). The relative expression data were analyzed employing the comparative CT (cycle threshold) method, with GAPDH serving as the endogenous control to normalize the expression levels. A comprehensive list of the primers utilized in this research is detailed in **Table S2**.

### Statistical analysis

We conducted all statistical analyses with R software, version 4.4.0, accessed through its official website (http://www.r-project.org). Significance was determined using a two-tailed P-value with a cutoff of less than 0.05.

## Results

### The MRGs landscape in breast cancer

**Figure 2:**
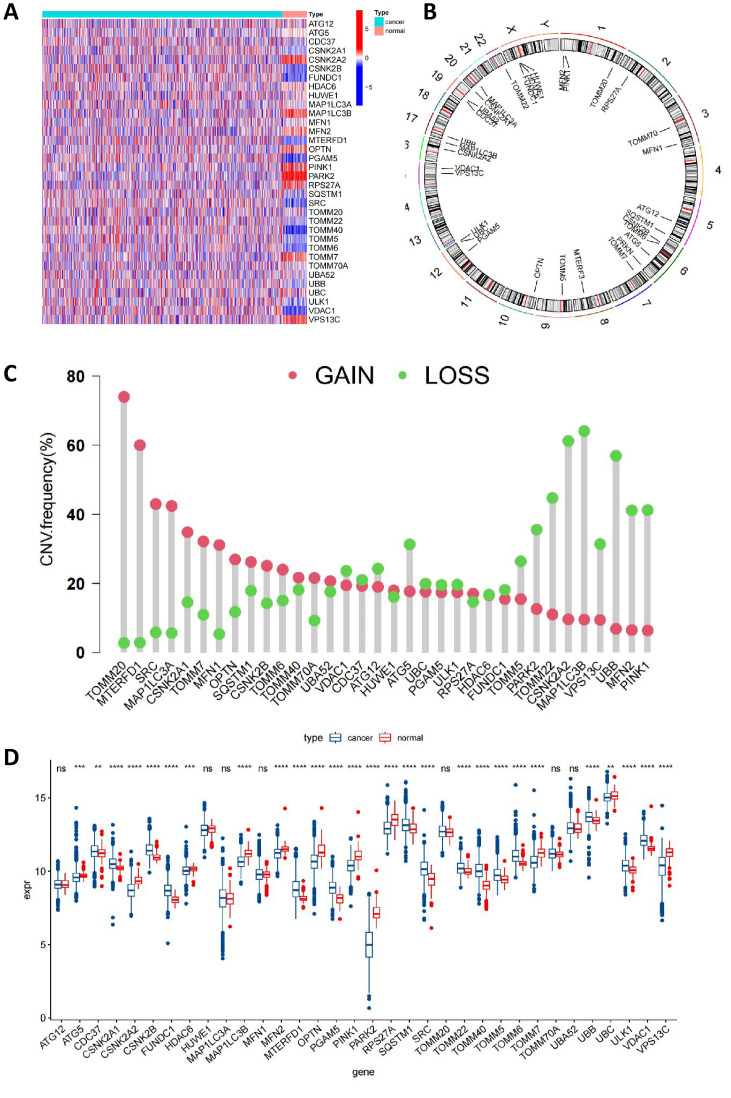
** The MRGs landscape in breast cancer.** (A) Comparison of MRGs between tumor and normal group; (B) Chromosomal locations of these genes; (C) The frequency of CNV gain and loss in MRGs; (D) mRNA expression of MRGs between tumor and normal tissues (t-test, **** *P* < 0.0001; *** *P* < 0.001; ** P < 0.01; * *P* < 0.05).

In this Comprehensive Study, We Integrated 34 MRGs into the TCGA Breast Cancer Database Cohort. Initially, we employed a heatmap (**Figure 2A**) to visually contrast the expression differences of the 34 genes between BC and normal tissues, highlighting the discordance in MRG expression between tumor and normal tissues. Subsequently, **Figure 2B** clearly delineated the specific chromosomal locations of these genes. Further, we investigated the CNV among these genes. Our analysis revealed a widespread presence of CNVs across all 34 mitophagy genes. Specifically, genes such as TOMM20, MTERFD1, SRC, MAP1LC3A, CSNK2A1, TOMM7, and MFN1 exhibited extensive CNV gains, while PINK1, MFN2, UBB, MAP1LC3B, CSNK2A2, and others demonstrated CNV losses (as shown in **Figure 2C** and **Table S3**). Subsequently, we conducted a detailed analysis of mRNA expression differences of MRGs between tumor and normal tissues (**Figure 2D**). The results indicated that, with the exception of ATG12, HUWE1, MAP1LC3A, MFN1, TOMM20, TOMM70A, and UBA52, the remaining genes all showed significant differential expression. These findings strongly suggest that MRGs play an essential role in the onset of BC. Additionally, we observed that in tumor tissues, many highly expressed MRGs are associated with CNV gains, such as MTERFD1, SRC, and MAP1LC3A. However, exceptions were noted, including TOMM20, which did not show significant differences in expression between tumor and normal tissues. This suggests that while CNVs can influence the expression of MRGs, they are not the sole determining factor.

### Identification of mitophagy subgroup in breast cancer

**Figure 3:**
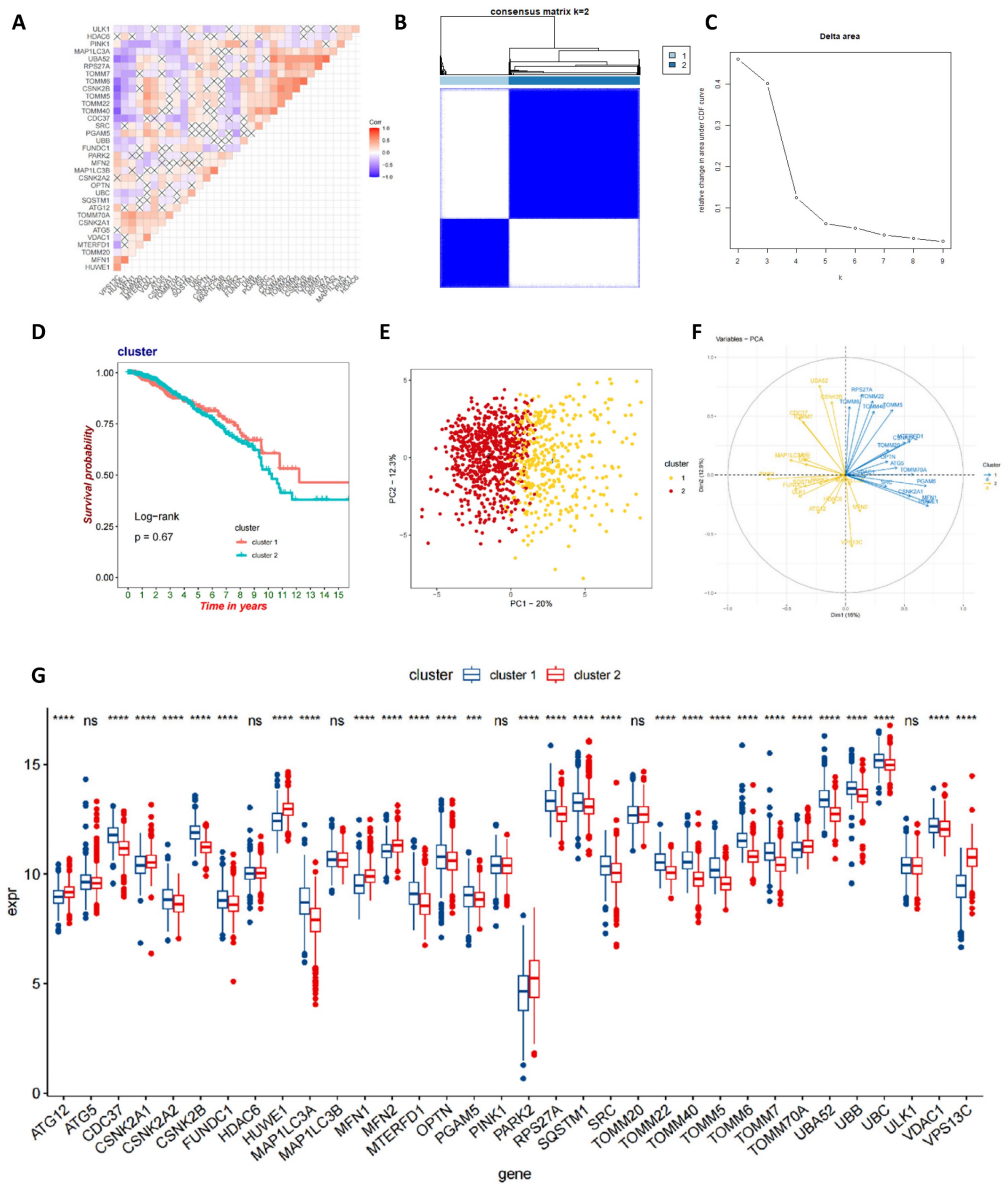
** Identification of mitophagy Subgroup in breast cancer.** (A) Correlation analysis of MRGs; (B, C) A consensus matrix heat map defining two clusters (k=2); (D) Kaplan-Meier analysis of three subtypes of OS. (E, F) PCA analysis of two mitophagy clusters. (G) The gene expression level of two mitophagy clusters (t-test, **** *P* < 0.0001; *** *P* < 0.001; ** P < 0.01; * *P* < 0.05).

Initially, we conducted an in-depth correlation analysis for these 34 genes (**Figure 3A**). To explore the expression patterns of MRGs in BC patients, we applied a consensus clustering algorithm, meticulously classifying the samples based on the expression profiles of these 34 genes. The clustering results indicated that when employing the k-means clustering algorithm with k set to 2, the patient population could be distinctly divided into two separate clusters, demonstrating a high degree of stability in this classification (**Figure 3B-C**). Furthermore, we performed Kaplan-Meier survival analysis on these two MRG subgroups and observed that patients in the second group had relatively poorer prognosis. However, it is important to note that the P-value from the log-rank test was 0.67, indicating that the survival difference between the two groups was not statistically significant (**Figure 3D**). To gain a more comprehensive understanding of the transcriptomic differences between these subgroups, we carried out principal component analysis (PCA). The results revealed that there indeed were significant differences in the transcriptomic profiles between the two subgroups (**Figure 3E-F**). Lastly, box plots and heatmap were used to compare the distribution of MRGs across different MRG subgroups in BC (**Figure 3G** and **Figure S1**).

### Characteristics of the biological behavior in mitophagy subgroups

**Figure 4:**
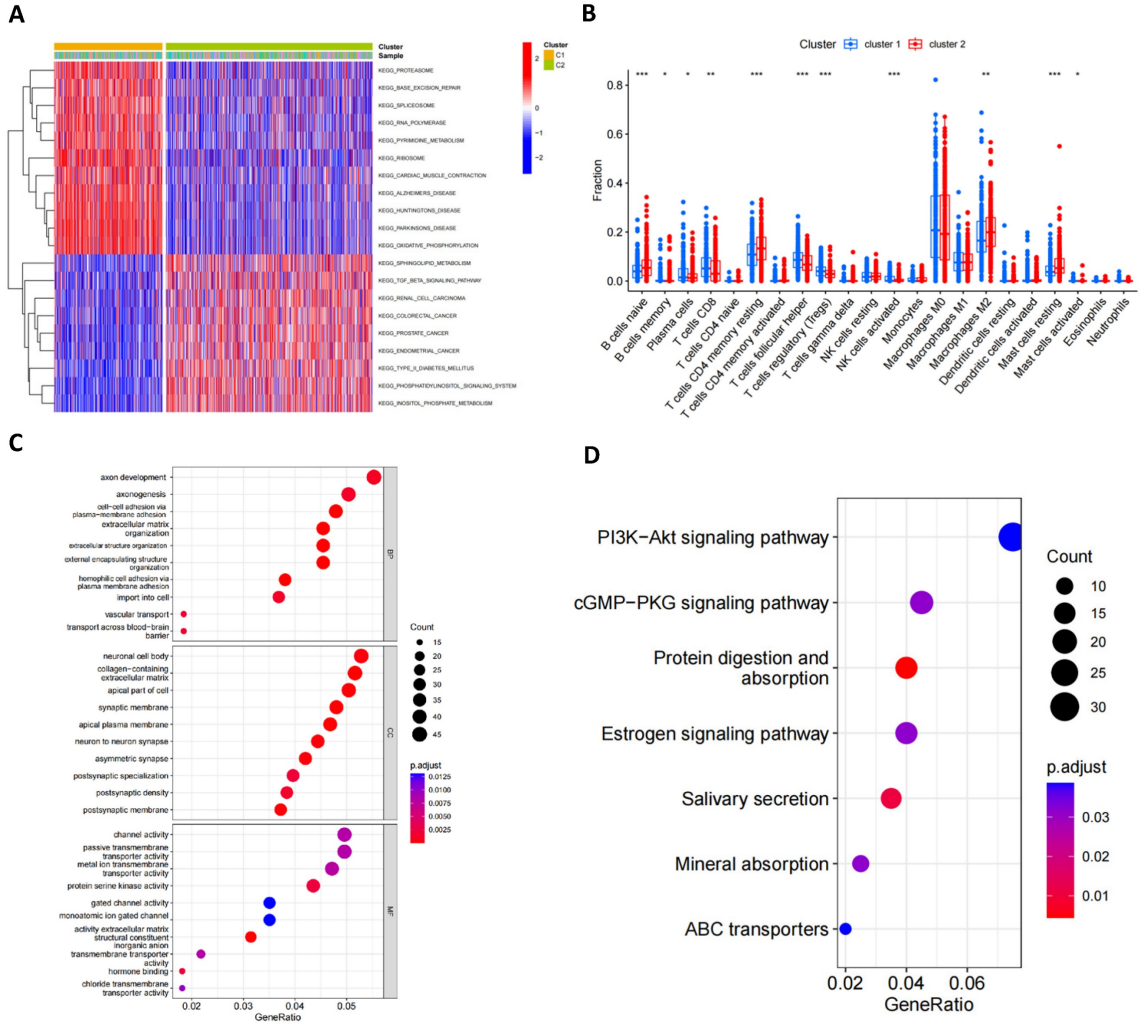
** Characteristics of the biological behavior in mitophagy subgroups.** (A) GSVA analysis of two mitophagy clusters. (B) Immune cell infiltration of two mitophagy clusters. (C, D) GO (C) and KEGG (D) enrichment analysis of DEGs among two mitophagy clusters (t-test, *** *P* < 0.001; ** P < 0.01; * *P* < 0.05).

GSVA analysis indicated significant statistical differences in the main biological processes enriched in the two subtypes (**Figure 4A; Table S4**). Cluster 1 was significantly enriched in biological processes such as proteasome, base excision repair, spliceosome, polymerase, metabolism, ribosome, cardiac muscle contraction, Alzheimer's disease, Huntington's disease, Parkinson's disease and oxidative phosphorylation, while cluster 2 was primarily enriched in the sphingolipid metabolism, TGF beta signaling pathway, renal cell carcinoma, colorectal cancer, prostate cancer, endometrial cancer, type 2 diabetes mellitus, phosphatidylinositol signaling system and inositol phosphate metabolism pathway. Furthermore, **Figure 4B** illustrated the infiltration levels of 22 immune cells across the two clusters. We found statistically significant differences in the infiltration of most immune cells between the two subgroups, with NK cells, T cells follicular helper, Tregs and CD8^+^ T cells infiltrating at a significantly higher rate in cluster 1 compared to cluster 2. However, cluster 2 exhibited greater immune cell infiltration, such as B cells naïve, CD4^+^ T cell memory resting, M2 macrophages and resting mast cells. Additionally, we identified 1013 mitophagy subtype-related DEGs (**Table S5**) and performed functional enrichment analysis (**Figure 4C, D**). GO analysis revealed that these MRG-related DEGs are involved in biological processes such as axon development, axon genesis, neuronal cell body channel activity, etc. KEGG pathway analysis indicated that these MRG-related DEGs are primarily associated with Pl3K-Akt signaling pathway, cGMP-PKG signaling pathway, protein digestion and absorption, estrogen signaling pathway, salivary secretion, mineral absorption and ABC transporters.

### Identification of gene subtypes in BC based on DEGs

**Figure 5:**
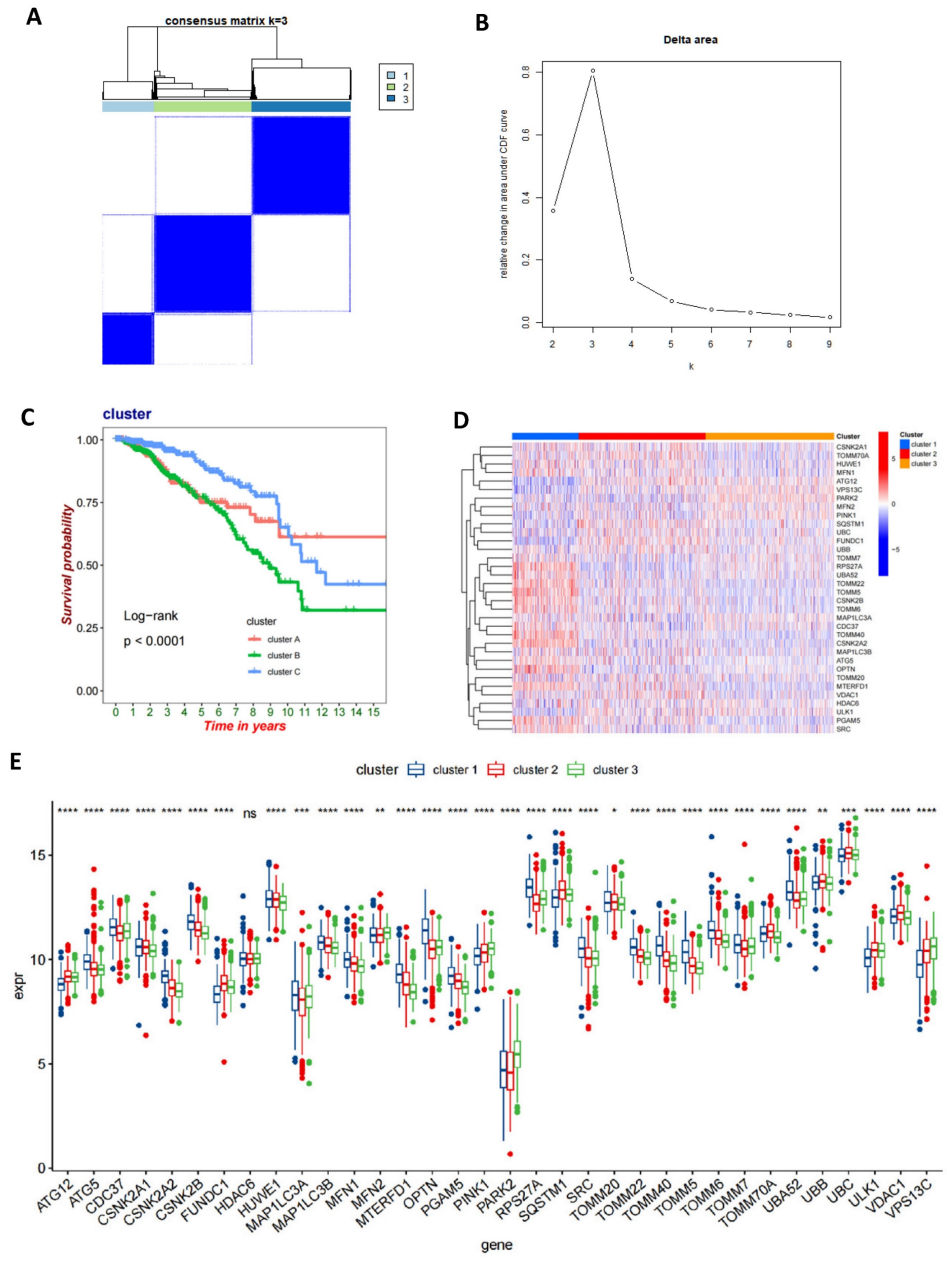
** Identification of gene subtypes in BC based on DEGs.** (A, B) A consensus matrix heat map defining three gene subtypes (k=3); (C) Kaplan-Meier analysis of three subtypes of OS. (D, E) The MRGs expression level of three gene subtypes (t-test, **** *P* < 0.0001; *** *P* < 0.001; ** P < 0.01; * *P* < 0.05).

We performed univariate Cox regression analysis on 1013 DEGs to evaluate their prognostic value in BC. Utilizing a cutoff criterion of a P-value less than 0.05, we selected 112 genes for further detailed analysis (**Table S6**). Subsequently, based on these prognostic-relevant genes, we applied a consensus clustering algorithm to categorize the cohort into three distinct genetic subtypes (**Figures 5A-B**). Furthermore, employing Kaplan-Meier survival analysis, we observed significant differences in OS among these three genetic subtypes (P<0.0001), with genetic cluster B exhibiting the poorest prognosis (**Figure 5C**). Following this, we analyzed the expression variances of MRGs across each genetic cluster and discovered significant differences in MRG expression among these clusters (**Figures 5D-E**).

### Construction of mitophagy-related prognostic risk score

**Figure 6:**
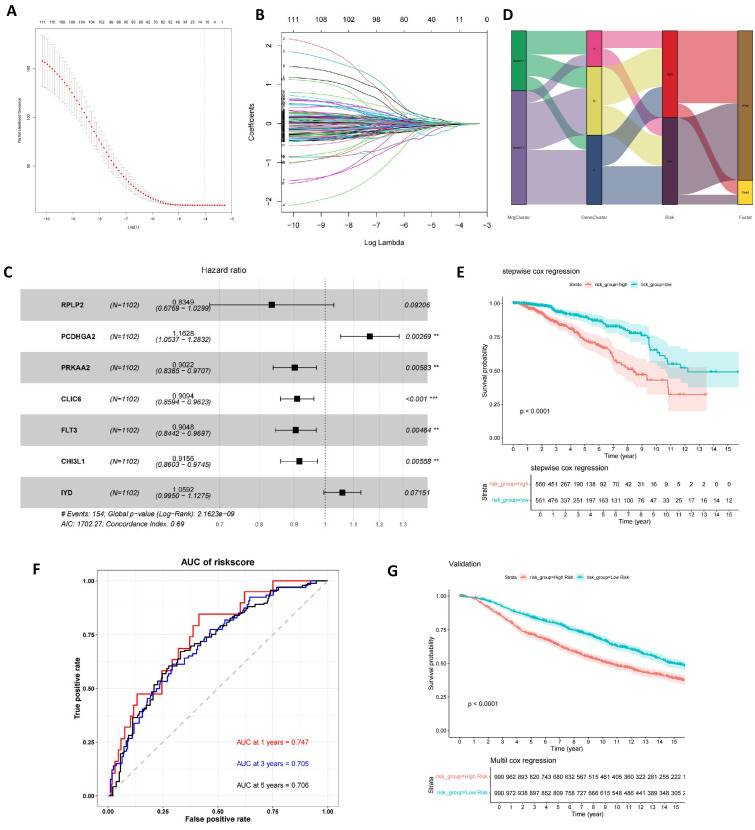
** Construction of mitophagy-related prognostic risk score.** (A, B) Lasso regression analysis on the prognosis-related genes; (C) Multivariate Cox regression analysis; (D) The sankey diagram of the sample distribution of two mitophagy clusters, three gene subtypes and two risk score groups. (E) OS analysis of two risk groups using Kaplan-Meier in the training cohort; (F) ROC curves to predict 1, 3, and 5-year OS according to the risk score in the training cohort; (G) OS analysis of two risk groups using Kaplan-Meier in the validation set (t-test, *** *P* < 0.001; ** P < 0.01; * *P* < 0.05).

Utilizing these prognostic-relevant genes, we selected the most critical prognostic factors through Lasso regression and stepwise multivariate Cox analysis. Seven overall survival (OS)-related genes (RPLP2, PCDHGA2, PRKAA2, CLIC6, FLT3, CHI3L1, and IYD) were identified and retained for further analysis (**Figures 6A-C** and **Table S7**). Patients were categorized into low- and high-risk groups based on the median risk score. A Sankey plot illustrated the distribution of samples across two mitophagy clusters, three genetic clusters, and two risk score groups (**Figure 6D**). Kaplan-Meier survival analysis of the two risk groups revealed that patients in the low-risk group had significantly better OS compared to the high-risk group (log-rank test, P<0.0001; **Figure 6E**). Moreover, the 1-year, 3-year, and 5-year area under the curve (AUC) values for the risk score model were 0.747, 0.705, and 0.706, respectively (**Figure 6F**). In the validation set, the Kaplan-Meier survival analysis of the two risk groups also showed a significant difference, with patients in the low-risk group having significantly better OS compared to the high-risk group (log-rank test, P<0.0001; **Figure 6G**). Finally, we found that the risk scores among the three gene clusters are significantly different. Cluster B has the highest risk score, followed by cluster A, with cluster C having the lowest score. There are also differences in risk scores between the two MRG clusters (**Figure S2 A-B**).

### Development and validation of a prognostic nomogram for breast cancer

**Figure 7:**
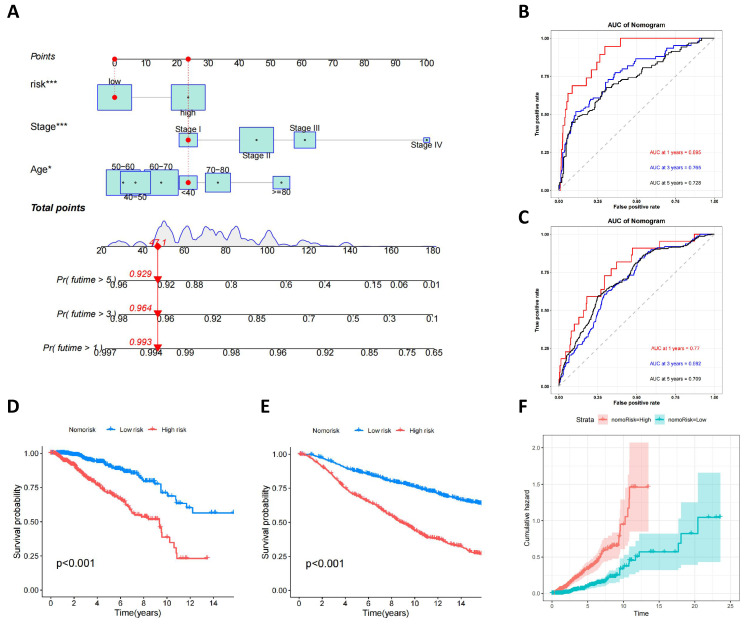
** Development and Validation of a Prognostic Nomogram for Breast Cancer.** (A) A nomogram used to predict BC OS; (B) ROC curves to predict 1-, 3-, and 5year OS according to the nomogram in the training cohort; (C) ROC curves to predict 1-, 3-, and 5year OS according to the nomogram in the verification cohort; (D) OS analysis of two nomorisk groups using Kaplan-Meier in the training cohort and validation cohort; (E) Cumulative hazard of two nomorisk groups in the training cohort.

To enhance clinical utility and improve the accuracy of the prognostic model, clinical and pathological parameters, including age and staging, were incorporated into the aforementioned prognostic risk model to construct a more comprehensive nomogram for predicting OS in BC (**Figure 7A**). The model was validated for its good discriminatory ability. In the training set, the AUC values for 1, 3, and 5 years were 0.895, 0.765, and 0.728, respectively (**Figure 7B**), the C-index curve shows that the model has good consistency (**Figure S3A**), and the AUC values for 1, 3, and 5 years in the validation set were 0.77, 0.692, and 0.709, respectively (**Figure 7C**). The calibration curve suggested that the model had good correction ability (**Figure S3B-C**). We utilized the obtained nomorisk to divide the samples into high-risk and low-risk groups. In both the training and validation groups, the survival of the low-risk group was significantly better than that of the high-risk group (**Figure 7D-E**). As time increases, the cumulative hazard of the high-risk group is higher than that of the low-risk group both in the training set (**Figure 7F**) and validation set (**Figure S3D**).

### Characteristics of the TME, mutation, CSC Index, and drug susceptibility analysis in the high and low risk groups

**Figure 8:**
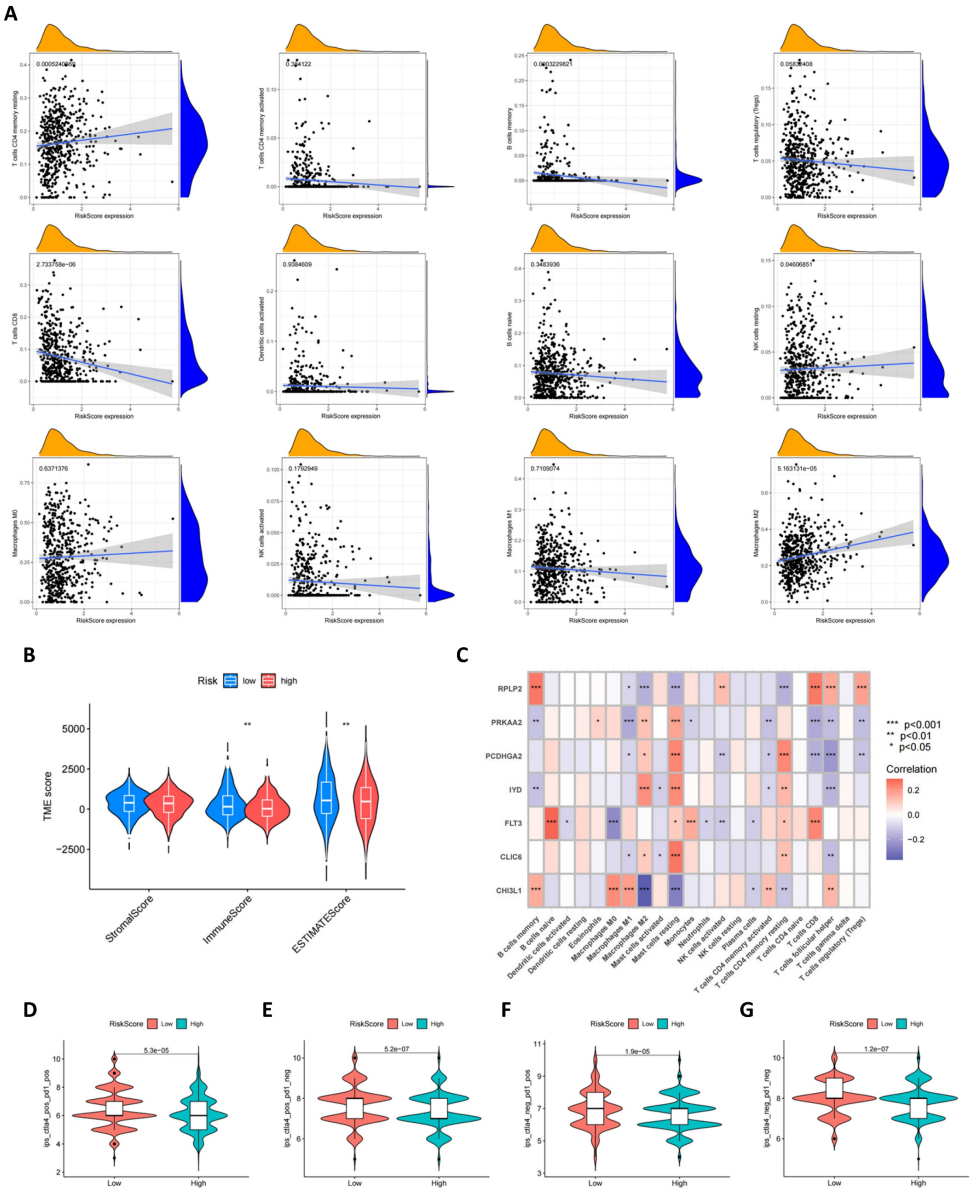
** TME and immune checkpoint characteristics in both risk score groups.** (A) Association of risk score with immune cell infiltration; (B) Association between risk score and TME score; (C) Association between immune cell infiltration and seven genes in the risk score model; (D-G) Immunotherapy effect in the low- and high-risk groups (t-test, *** *P* < 0.001; ** P < 0.01; * *P* < 0.05).

Using the CIBERSORT algorithm, we conducted an in-depth analysis of the correlation between risk scores and the infiltration of various immune cells. **Figure 8A** visually presents the correlation patterns between risk scores and immune cell populations, where risk scores exhibit a negative correlation trend with cell types such as macrophages M1, memory B cells, Tregs, CD8^+^ T cells, etc. A positive correlation is observed with cell types such as macrophages M2, CD4^+^ memory T cells, etc. Furthermore, as shown in **Figure 8B**, compared to high-risk scores, low-risk scores are associated with higher immune scores, which may reflect the degree of immune response activity. Subsequently, we further explored the relationship between the seven specific genes in the model and immune cell populations. Through detailed analysis, we found that these genes have significant correlations with most immune cell types, as shown in **Figure 8C**, revealing their important role in regulating immune cell functions. In addition to gene analysis, we also focused on the differences in immune therapy outcomes between the two risk groups. The results from **Figures 8D-G** reveal differences in immune therapy responses among different risk groups, with demonstrating higher therapeutic effects in low-risk groups.

**Figure 9:**
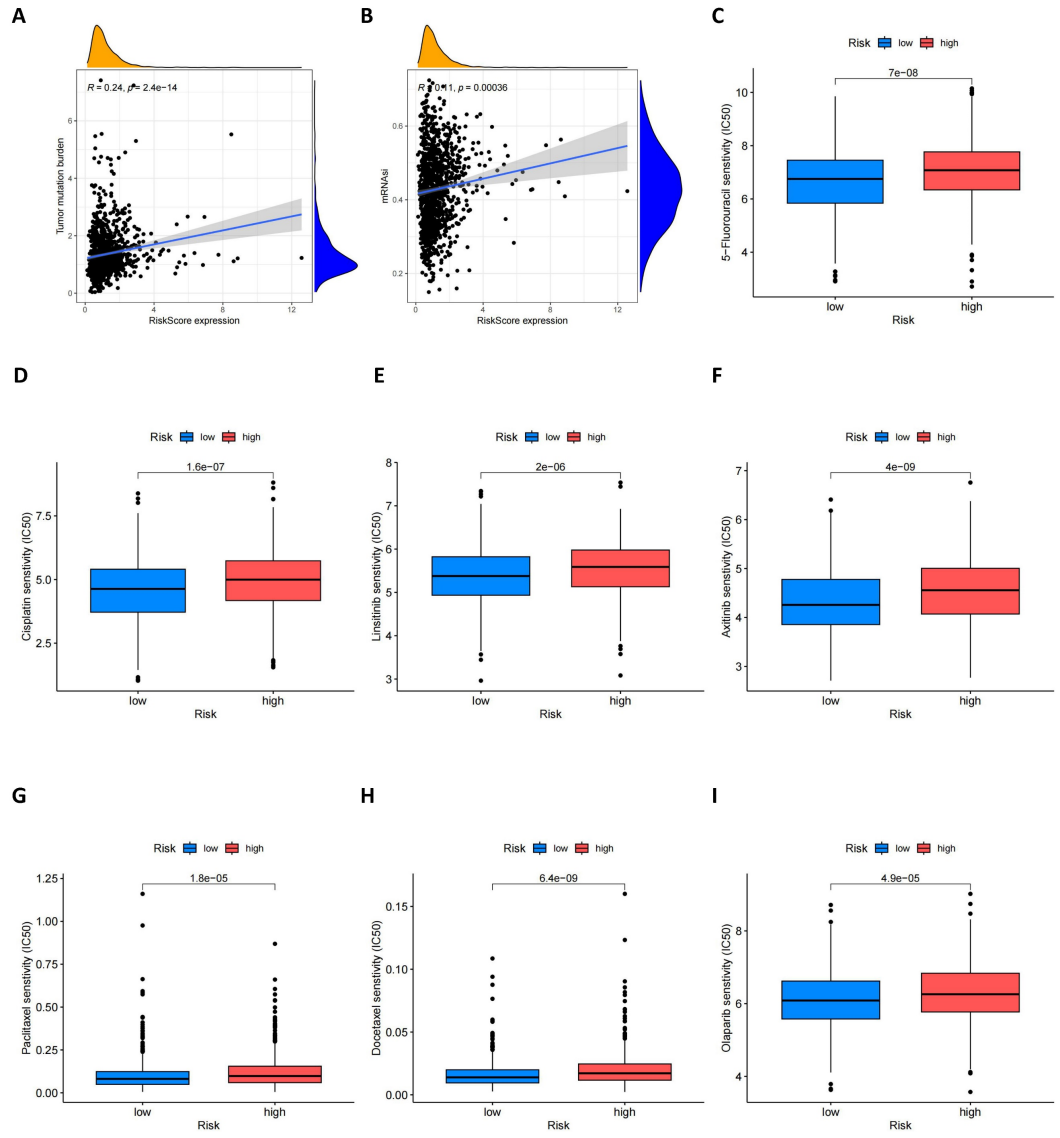
** TMB, CSC index and drug susceptibility analysis among two risk_score groups.** (A) Correlation between risk score and TMB; (B) Correlation between risk score and CSC index; (C-I) Correlation between risk score and drug susceptibility.

Furthermore, we explored the relationship between risk scores and tumor mutational burden (TMB). As shown in **Figure 9A**, there is an evident positive relationship between risk scores and TMB, which may provide new insights for future immunotherapy strategies. At the same time, we assessed the potential link between cancer stem cell (CSC) index values and risk scores. **Figure 9B** shows that risk scores are positively correlated with the CSC index, indicating that BC cells with high-risk scores may possess more pronounced stem cell characteristics, which is significant for understanding tumor recurrence and metastasis. Lastly, we evaluated the sensitivity of high-risk and low-risk groups of BC patients to different commonly used therapeutic drugs. As shown in **Figures 9C-I**, most therapeutic drugs have lower IC50 values in the low-risk population, including 5-fluorouacil, cisplatin, linsitinib, Olaparib and paclitaxel etc., providing important reference for the formulation of clinical treatment strategies.

### Single cell verification of the distribution of prognostic genes in breast cancer

**Figure 10:**
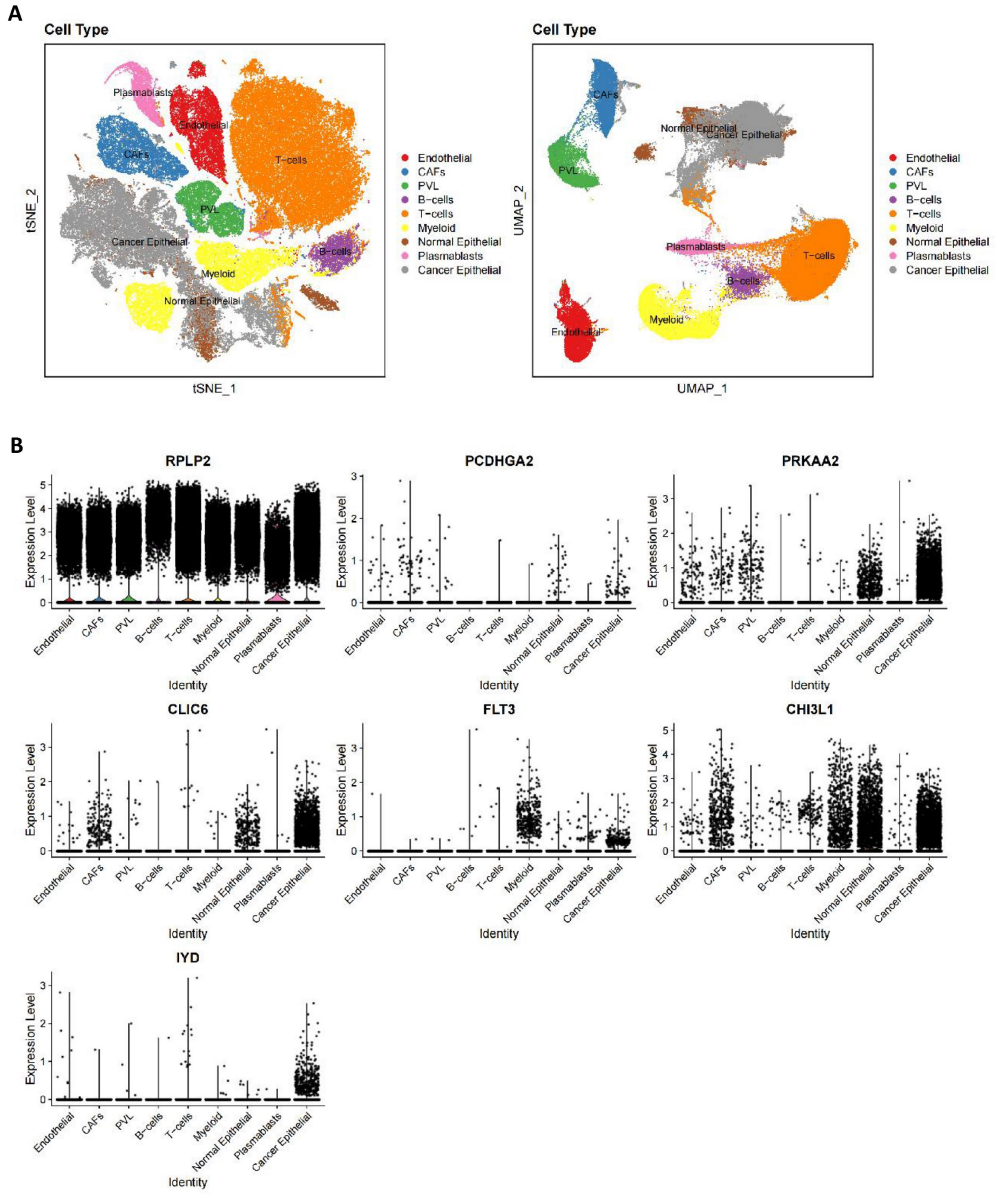
** Single cell verification of the distribution of prognostic genes in breast cancer.** (A) tSNE and UMAP projections of breast cancer cells in GSE176078. Different cell types are indicated by unique colors; (B) Delineating the distribution of key genes in cell subsets.

According to the cellular annotation results provided by the data contributors, cells within the breast cancer (BC) tissue were classified into nine cellular subpopulations: endothelial cells, cancer-associated fibroblasts (CAFs), perivascular-like (PVL) cells, B cells, T cells, myeloid cells, normal epithelial cells, plasmablasts, and cancer epithelial cells (**Figure 10A**). RPLP2 is expressed at relatively high levels across nearly all cell types. PCDHGA2 exhibits low expression across all cell clusters, with primary expression in CAFs and epithelial cells. PRKAA2 is predominantly expressed in epithelial cells, particularly cancer epithelial cells. CLIC6 is also primarily expressed in cancer epithelial cells, followed by CAFs and normal epithelial cells. FLT3 is mainly observed in cancer epithelial and myeloid cells, with minimal expression in normal epithelial cells. CHI3L1 is primarily expressed in epithelial cells, followed by CAFs and myeloid cells, and IYD is specifically expressed in cancer epithelial cells, with minimal expression in other cells (**Figure 10B**).

### Verification of the expression level of seven mitophagy related genes in the risk model

**Figure 11:**
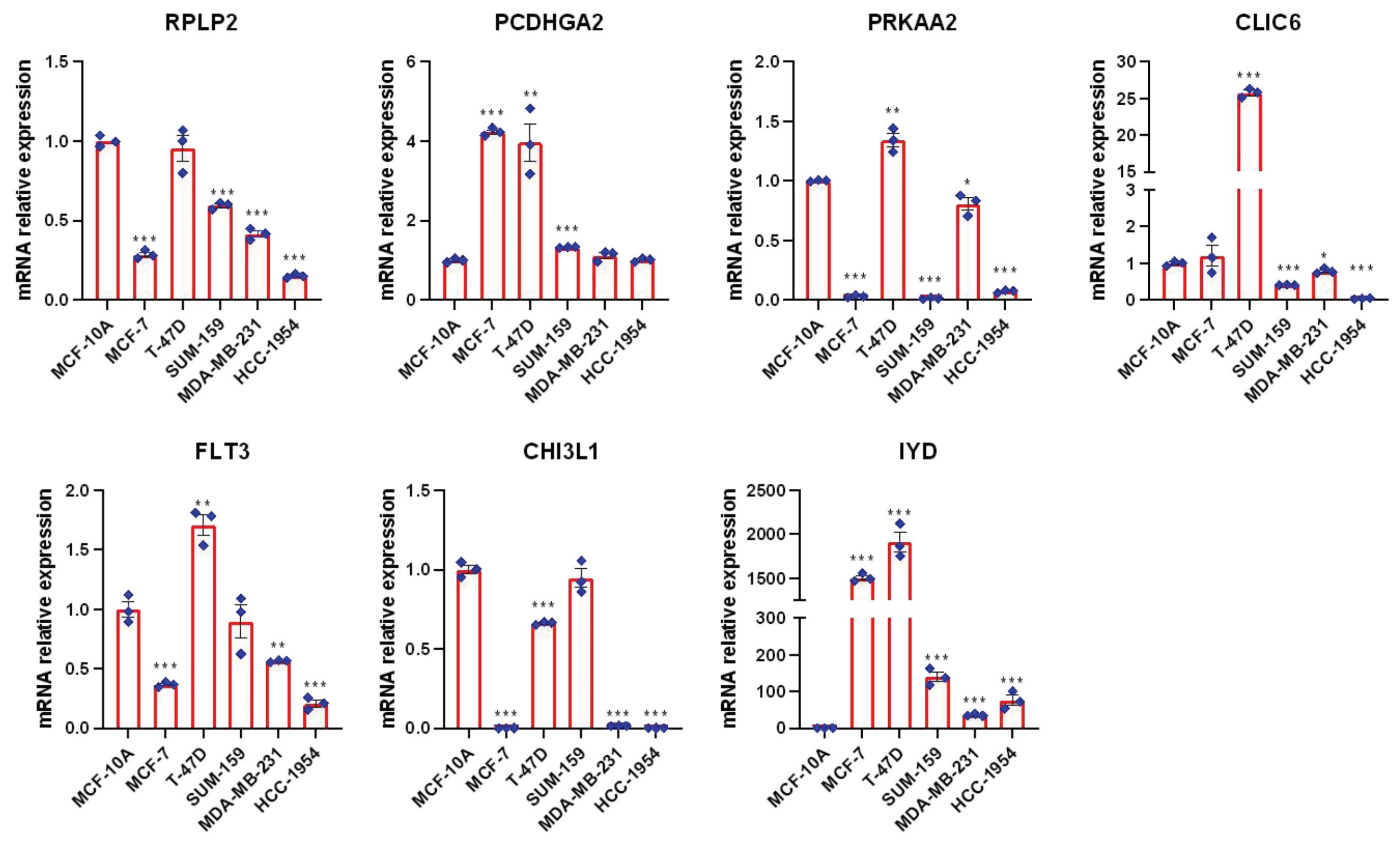
RT-PCR was used to compare mRNA levels of seven prognostic mitophagy-related genes in breast cancer cells and normal mammary epithelial cells (t-test, *** *P* < 0.001; ** *P* < 0.01; * *P* < 0.05).

To further validate the expression profiles of these genes in BC, we employed the reverse transcription quantitative polymerase chain reaction (RT-qPCR) technique to measure their expression levels across five different BC cell types as well as one normal mammary epithelial cell line (MCF-10A). As shown in **Figure 11**, compared to the normal mammary epithelial cells MCF-10A, these prognostic genes exhibit distinct expression patterns in BC cell types, As shown in Figure 11, compared to MCF-10A, the expression levels of RPLP2, PRKAA2, CLIC6, FLT3, CHI3L1, etc., have decreased in most breast cancer (BC) cells, while the expression levels of PCDHGA2 and IYD have been significantly upregulated, especially IYD, which is expressed very little in normal mammary epithelial cells, consistent with our previous single-cell results.

## Discussion

Despite progress in cancer research, BC continues to pose a global health challenge, necessitating further investigation and the development of innovative therapies^26^. Due to its complexity, heterogeneity, and significant individual variability, personalized diagnostic and treatment approaches are required^27^,^28^. Our study provides a comprehensive summary of the expression levels of MRGs, CNVs, immune infiltration, TME, CSCs, and drug sensitivity in BC. Based on 34 MRGs, we classified samples into two mitochondrial autophagy subtypes and distinguished three genetic subtypes using DEGs. Using GSVA and GSEA analyses, we examined the biological processes and immune cell infiltration between the two subtypes, revealing that cluster 1 is predominantly enriched in NK cells, T cells follicular helper, Tregs and CD8^+^ T cells, among others. Additionally, cluster 2 exhibits more B cells naïve, CD4^+^ T cell memory resting, M2 macrophages and resting mast cells. We identified three genetic subtypes and constructed a risk score model predictive of OS in BC. To enhance the clinical utility of the model, we incorporated clinical and pathological features to build a nomogram and validated its predictive performance. There are statistically significant differences in prognosis, mutations, TME, CSC index, and drug sensitivity between patients with low and high-risk scores. Our findings indicate that MRGs can be used to assess the prognostic significance and response to immunotherapy in BC.

At present, the integration of immunotherapy with chemotherapy embodies an innovative treatment strategy. In contrast to other solid malignancies, breast cancer (BC) demonstrates reduced immunogenicity and a diminished mutational load. Nonetheless, certain clinical trials have detected indications that anti-PD1/PD-L1 therapies may act in concert with chemotherapy^29^-^31^, with additional studies hinting at the possible advantages of immunotherapeutic interventions^32^. Our research indicates that the cohort with a lower risk profile might achieve superior results from immunotherapy, implying that their scores could be instrumental in evaluating the risks inherent to such treatment modalities.

Mitochondria, known as the cell's "powerhouses", are crucial for cell survival and death, primarily generating energy through the TCA cycle and oxidative phosphorylation. Dysfunctional mitochondria accumulation is linked to diseases like heart failure, Alzheimer's, and cancers. The mechanisms of mitophagy's impact on tumor growth and the immune environment are not fully understood. Mitophagy selectively targets mitochondrial proteins, such as those on the outer membrane involved in autophagy (e.g., LC3)^33^. and clears damaged mitochondria to maintain homeostasis. Key mitophagy proteins like PRKN^34^, BNIP3, BNIP3L, and FUNDC1^35^ play significant roles in regulating the process and influencing cellular functions^36^,^37^.

In previous studies, the prognosis for many BC patients was predicted solely based on clinical information^38^. For instance, staging systems are commonly utilized to forecast the prognosis of BC. However, the prognostic capacity of individual clinical parameters is often limited. Prior research has indicated a potential link between mitophagy and BC^39^. Consequently, our study focused on exploring mitophagy in BC and developed a model based on these findings. Our model holds promise for guiding BC treatment. Immunological analysis revealed varying immune scores across different risk groups, with these scores correlating to immune cell infiltration, suggesting that immunotherapy may be more efficacious in the low-risk cohort. As research into immunotherapy for breast cancer gains momentum, strategies such as inducing macrophage polarization also show great potential in cancer treatment^40^.

Our study also explored TMB and CSC across different risk groups. Previously, TMB has been established as a predictive biomarker for the efficacy of cancer immunotherapy across various types of cancer^41^. TMB has a strong corrective effect on the therapeutic outcomes and prognosis of cancer^42^-^44^. In our analysis, there were also significant differences in TMB and survival rates among different risk groups, suggesting that the mitochondrial autophagy pathway may be associated with tumor mutations. Similarly, CSC are associated with the response to immunotherapy. Targeting CSC may potentially prevent metastasis and improve survival rates in BC. Recent evidence suggests that mitochondrial autophagy is also involved in regulating cellular stemness and the rate of differentiation within tissues^45^. There is evidence that a reduction in mitochondrial autophagy can also modulate cellular stemness and regulate the rate of differentiation within tissues. Studies have found that mitochondria undergo asymmetric division in immortalized human mammary epithelial stem cells, with the newly formed, healthy mitochondria in these breast stem cells preferentially undergoing division^46^,^47^. In contrast, the mitochondria in non-stem cells are confined to the perinuclear region. In short, the senescence of mitochondria can lead to a reduction in cellular stemness^45^. Additionally, CSC are a source of chemoresistance in BC. Analysis of drug sensitivity has shown that the IC50 values for some commonly used chemotherapeutic drugs are lower in the low-risk group compared to the high-risk group. Therefore, targeting CSC therapy could potentially prevent metastasis and thus improve the survival of BC^48^. Finally, we explored the distribution of these genes in breast tissue samples through single-cell data analysis, which may provide indications for subsequent further research. This includes the distribution of these genes, their possible mechanisms, and interactions, among others. Thus, we believe that our research can offer assistance, potentially contributing to the resolution of certain cancer treatment strategies. This could lead to more effective personalized treatment plans for individuals, minimizing unnecessary side effects and providing new insights and perspectives for further investigation into its role in breast cancer.

Despite the initial slow pace of biomarker development, which may have been due to the complexity of requirements for tissue samples and sequencing platforms, the rapid advancement of genetic sequencing technologies in recent years has significantly enhanced our understanding of the differences in gene expression between various types of tumor cells. This has transformed our comprehension of cancer progression, the development of therapeutic strategies, and the assessment of patient prognoses^49^. The widespread adoption of genetic sequencing and the customization of immunotherapeutic approaches are ushering in an era of innovation, where cancer treatment will increasingly be tailored to individual needs, and we will ultimately triumph over cancer.

Despite these advancements, our research has certain limitations. We utilized public databases to construct and validate our model framework. However, incomplete clinical data for some patients necessitated the exclusion of a significant amount of individual data, which may have influenced the results. Consequently, there is an urgent need for prospective studies to assess the clinical utility of this model for patients with breast cancer. Additionally, our findings were explored through single-cell sequencing, but limitations in sequencing depth and analytical conditions, coupled with the absence of comprehensive pre-and post-treatment control samples, such as single-cell data before and after therapy, hindered a more detailed analysis and classification. This includes exploring the impact of treatment and drug sensitivity. Lastly, the lack of a detailed database on immunotherapy for breast cancer impeded our ability to further confirm the prognostic significance of the model in the context of immunotherapy. Ultimately, the specific mechanisms of action of these genes, their interactions with each other, and potential associations with immune cells may require comprehensive functional experiments for clarification.

## Conclusion

In our research, we identified seven new mitophagy genes associated with prognosis through an in-depth analysis of public databases. Patients with lower-risk profiles showed improved survival, leading to the development of a prognostic nomogram model that integrates these genes along with key clinical parameters like age and stage. This model offers robust predictability for breast cancer patient survival, enhancing our understanding of the disease's progression. It paves the way for personalized treatment strategies and potential clinical benefits through precision medicine. As our comprehension of mitophagy's role in breast cancer deepens, we anticipate the advancement of more accurate and effective treatment approaches, providing patients with better therapeutic options and survival prospects.

## Supplementary Material

Supplementary figures.
