# Supplementary material for: Exploring the role of mitophagy-related genes in breast cancer: subtype classification and prognosis prediction
Source: Int J Med Sci. 2024 Oct 14;21(14):2664–82. doi: 10.7150/ijms.100785 (PMC11539391; doi:10.7150/ijms.100785)
Supplement: Supplementary file 1 — Supplementary figures. [file ijmsv21p2664s1.pdf]

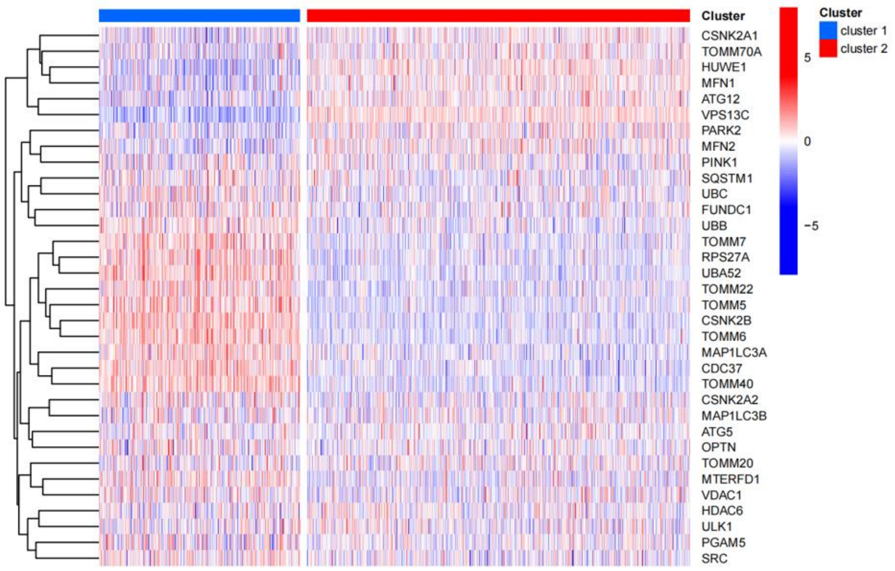

Figure S1. The MRGs expression level of two clusters.

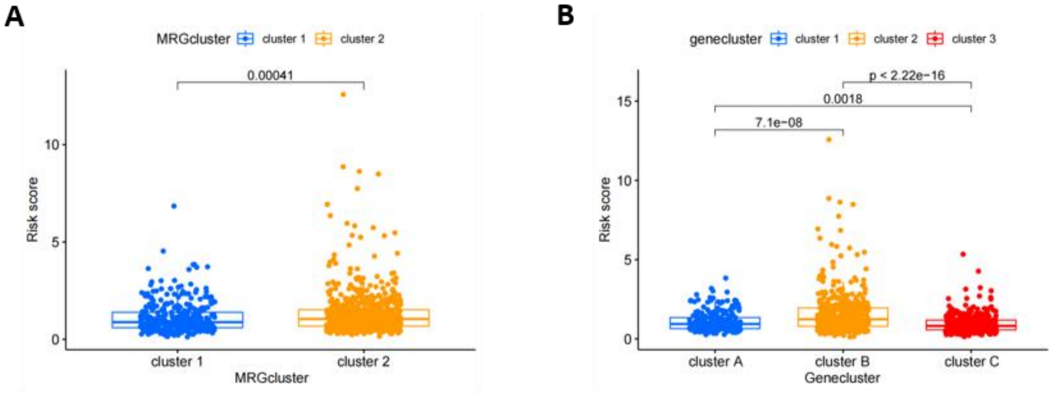

Figure S2. Construction of mitophagy-related prognostic risk score

The difference of risk score among two mitophagy clusters (A) and three gene subtypes (B)

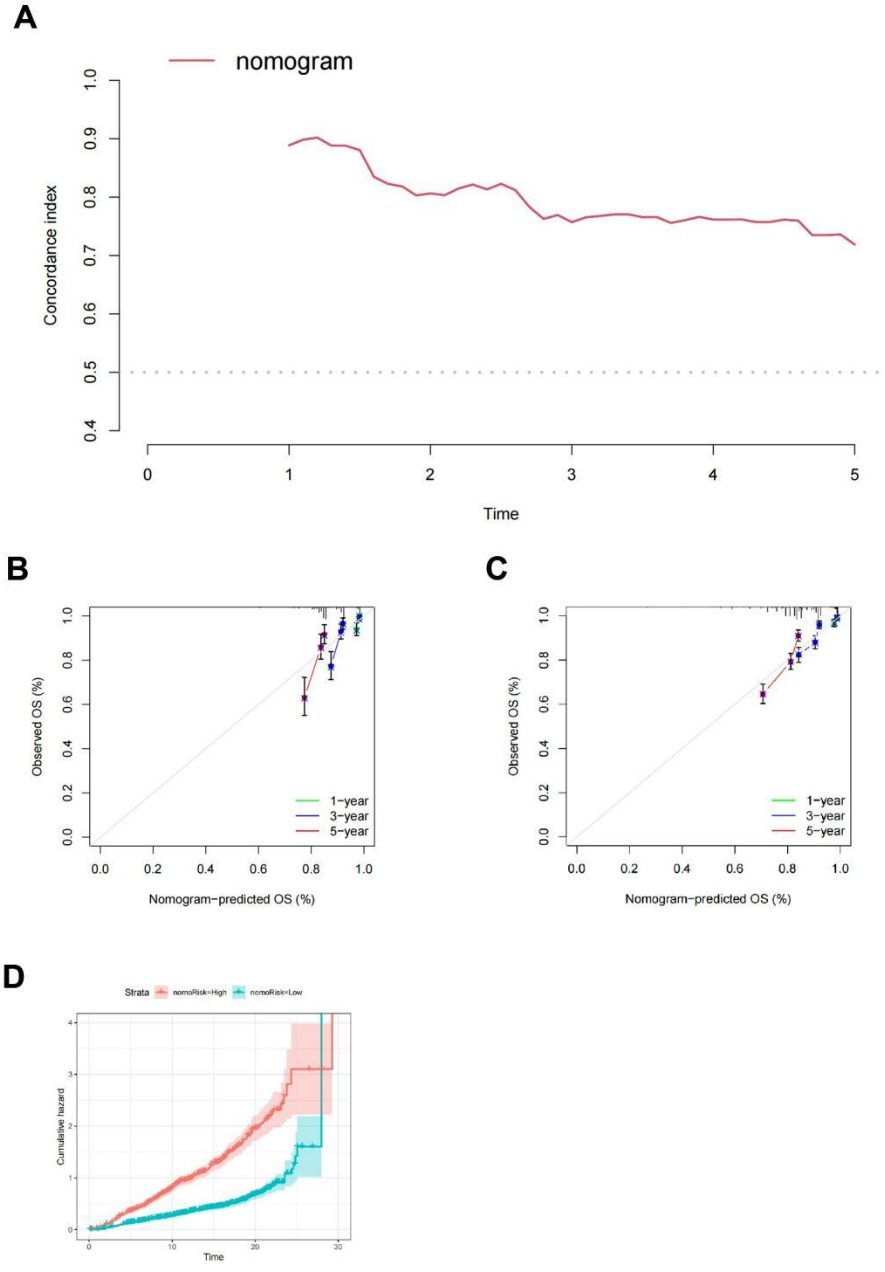

Figure S3. Development and Validation of a Prognostic Nomogram for Breast Cancer
